# Oral manifestations of morphea en plaque: Case report

**DOI:** 10.1016/j.amsu.2021.102891

**Published:** 2021-10-09

**Authors:** Samir Mainassara Chékaraou, Rajae El Gaouzi, Bouchra Taleb

**Affiliations:** a Oral Surgery Resident, Faculty of Dental Medicine-Rabat, Mohammed V University of Rabat, Morocco; b Oral Surgery, Faculty of Dental Medicine-Rabat, Mohammed V University of Rabat, Morocco

**Keywords:** Morphea en plaque, Oral manifestations, Oral cares

## Abstract

**Introduction:**

Morphea, or localized scleroderma, is an inflammatory disease that leads to sclerosis of the skin and underlying tissues due to excessive collagen deposition. Its etiology remains elusive. Morphea also affects oral and perioral tissues, the most common clinical manifestations being facial skin and tongue rigidity. Below, we present a case of morphea with oral manifestations.

**Case presentation:**

A 17-year-old patient was referred to our department of oral surgery by her dermatologist for possible oral morphea involvement. She presented pigmented skin lesions involving the right labial-chin region. On palpation, the skin of her perioral was sclerotic. the panoramic radiograph showed a shortening of the roots of the 46/47 with a developmental delay of the 48 compared to the 38. A bone biopsy was performed between 46/47 and distal to 48. histological examination showed bone tissue with fibrous and collagenous reorganization in favor of scleroderma.

**Conclusion:**

The management of plaque morphea is multidisciplinary. The role of the dentist is very important to avoid any oral complications.

## Revised Highlights

• “Morphea en plaque”is a rare condition involving in oro-facial area.
• Its etiology remains elusiveand its diagnosis requires a careful clinical, radiographic and histological examination.
• Themanagement of plaque morphea is multidisciplinary. The role of the dentist is very important to avoid any oral complications.

## 1 Introduction

‘Morphea en plaque’, also known as circumscribed morphea or localized scleroderma, is an inflammatory disease that causes sclerosis of the skin and underlying tissue due to excessive collagen deposition [1]. It is characterized by small violaceous skin patches or larger skin patches that indurate and cause loss of hair and sweat gland function [2]. The etiology of morphea remains controversial. Multiple mechanisms have been implicated, such as autoimmunity, infection, drugs, radiation and microchimerism [3]. they are classified into five sub-groups: plaque-like, generalized, bullous, linear (including en “coup de sabre”), and deep [4]. Morphea also affects oral and perioral tissues, the most common clinical manifestations being facial skin and tongue rigidity.

Below we report a case of oral and jaw manifestations of morphea en plaque, in line with the SCARE Criteria [5].

## 2 Patient and observation

**Fig. 1:**
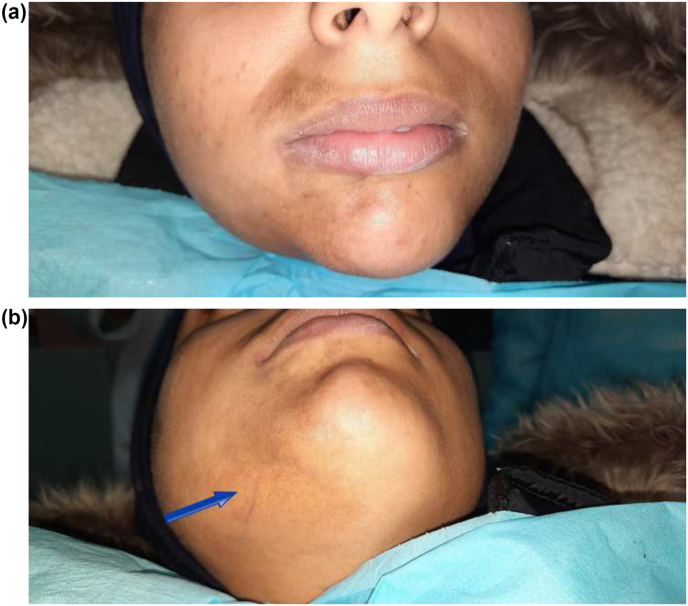
(A) extraoral view showing sclerotic lesions on the right area of the face; (B) extraoral view showing the telangiectasia on skin.

A 17-year-old patient caucasian origin was referred by her dermatologist to our oral surgery department for possible oral morphea involvement. On her personal history; the patient reported suffering from "morphea en plaque" for nine years without family history. Extraoral examination revealed a pigmented skin lesion affecting the right labial-chin region (Fig. 1A) with telangiectasia (Fig. 1B). On palpation, the skin of its perioral was sclerotic. No limitation of mouth opening was also observed. Intraoral examination showed no cortical swelling with normal tongue. The cold pulp vitality and percussion tests performed on the 46/47 were negative.

**Fig. 2:**
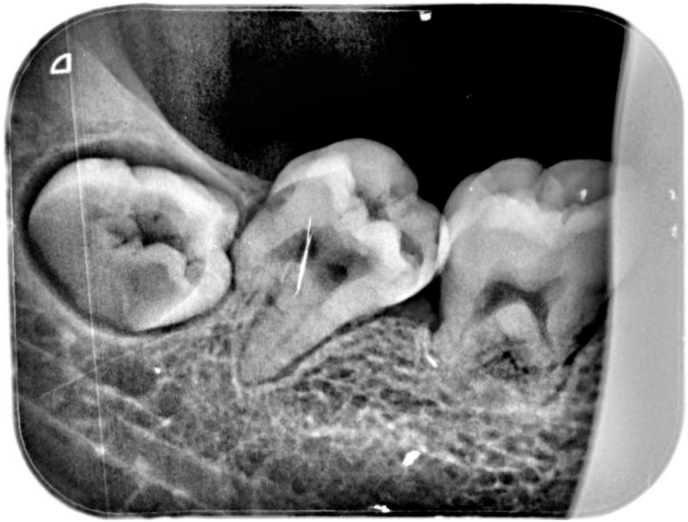
Periapical X-ray showing shortened roots with mesial enlargement of periodontal ligament of 47.

**Fig. 3:**
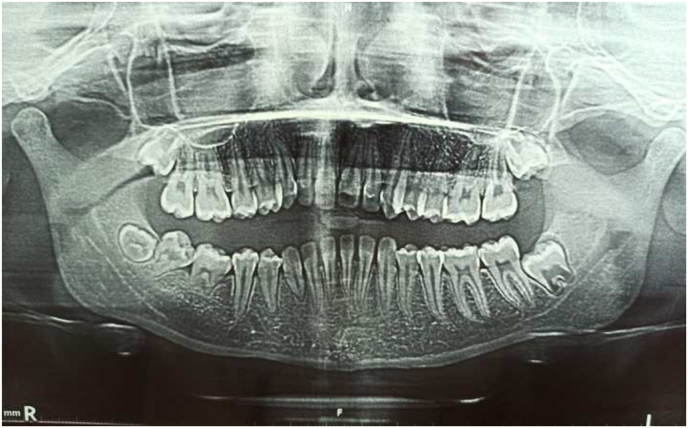
Panoramic radiography showing the shortened root of 47/48 and an abnormal development of 48.

The panoramic radiography showed a shortened root of 46/47 with a developmental delay of 48 compared to 38 (Fig. 2). The periapical x-ray confirmed the root shortening with a mesial enlargement of the periodontal ligament at tooth 47 (Fig. 3).

**Fig. 4:**
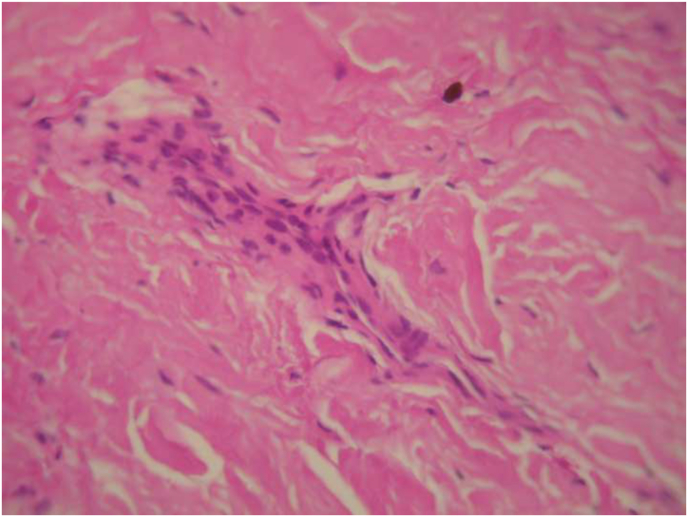
Anatomo-pathological examination of the lesion (hematoxylin-eosin staining. original magnification×100) showing bone tissue with fibrous and collagenous reorganization.

A bone biopsy was performed between 46/47 and distal to 48. Histological examination showed bone tissue with fibrous and collagen reorganization in favor of scleroderma (Fig. 4).

In collaboration with her dermatologist, the patient started a treatment based on corticosteroids (prednisolone 1 mg/kg/d), Vitamin D and folic acid (5 mg).

Regular follow-up every six months was introduced as well as instructions on motivation for oral hygiene instructions were given to the patient to avoid possible periodontal complications.

## 3 Discussion

"Morphea en plaque", or localized scleroderma, is an inflammatory condition that results in sclerosis of the skin and soft tissue, which is associated with negative impact on function, cosmesis, and life quality [4]. This condition is distinguished from systemic scleroderma by the absence of Raynaud's phenomenon and systemic organ involvement [1]. Morphea can present several clinical and course presentations. Although in the vast majority of cases there is no internal organ involvement, the fibrotic process can spread and affect deep structures such as the hypodermis, muscles, joints and bones, resulting in severe disfigurement and morbidity [3].

They are classified into five sub-groups: plaque-like as in our case, generalized, bullous, deep and linear [4]. Linear scleroderma, a subtype of morphea, is strongly associated with Parry–Romberg syndrome (facial hemiatrophy) [6].

The incidence of localized scleroderma is estimated to be 2.7 cases per 100,000 persons per year, and the prevalence of orofacial involvement in localized scleroderma is reported to be 7% [7,8]. According to the study by Prasada and al, oral manifestations would be found more in young patients (11.5 years) and in the majority of women (78%) such as in our case [4]. Oral manifestations are rare, and are rarely described in the literature. These manifestations can be characterized by dental anomalies and root development defects, root atrophy, malocclusions, limitations of the mouth opening due to sclerosis of the masticatory muscles. Problems of the temporo-mandibular joint (arthritis), alveolar bone resorption and gingival recessions have also been reported [4,9,10]. Speech and swallowing disorders can also be present as complications, especially in the rigidity of the tongue caused by this pathology [11]. In our case, anomalies are limited to root atrophy where we have a shortening of the roots of 46 and 47 with a delay in development of 48 compared to 38, no other perturbations have been noticed.

Radiographically, an increase in the thickness of the lamina dura has been evident and, is often more pronounced in the posterior teeth such as in our case [12]. Bone resorption has been observed at the angle of the mandible, as well as the coronoid process and the condyle, probably caused by pressure atrophy secondary to ischemia [13]. The resorption could be so severe as to cause pathologic fracture of the mandible [14].

In our patient no resorption has been reported despite of bone involvement in addition to the signs in the right lip and chin, confirmed by biopsy (Fig. 4).

There is no established protocol for the treatment of morphea en plaque [12]. Medical treatments include topical, intralesional, or systemic glucocorticoids, vitamin E, vitamin D3, retinoid, penicillin, griseofulvin, and interferon-alpha.

For those with a more severe and progressive disease, treatment includes methotrexate, corticosteroids, cyclophosphamide, and azathioprine [6].

Odontological management is done on a case-by-case basis by the dentist. It begins with motivation and the maintenance of adequate oral hygiene to limit gingival recessions. For the management of xerostomies, sialogogues can be prescribe (pilocarpine) or foods that stimulate salivation (menth candy).

In our patient, the approach was to motivate oral hygiene and follow-up to avoid any oral complications.

Patients with limited range of motion and mouth opening can benefit from regular physical and occupational therapy to maintain range of motion and to minimize or delay contractures. Rarely, patients may benefit from bilateral commissurotomy to increase the width of their mouth [15].

It is recommended that patients with localized scleroderma visit the dentist regularly to ensure the maintenance of good oral health care [11].

## 4 Conclusion

Oral manifestations of morphea en plaque are quite rare. They require multidisciplinary care and regular patient monitoring. The role of the dentist is very important for the treatment and the stability of oral lesions.

## Funding source

No source has funded this manuscript.

## Ethical approval

All the authors have read and complied with the policy of the journal on ethical consent.

## Consent

Written informed consent was obtained from the patient for publication of this case report and accompanying images. A copy of the written consent is available for review by the Editor-in-Chief of this journal on request.

Provenance and peer review Not commissioned, externally peer-reviewed.

## Author contribution

Dr MAINASSARA CHEKARAOU Samir and Rajae ELGAOUZI designed the concept, analyzed and interpreted the findings, wrote and reviewed the final paper under the supervision of Prof Bouchra TALEB.

## Guarantor

Mainassara Chékaraou Samir.

## Declaration of competing interest

The authors declare that they have no conflict of interest.

## Annals of medicine and surgery

The following information is required for submission. Please note that failure to respond to these questions/statements will mean your submission will be returned. If you have nothing to declare in any of these categories then this should be stated.

## Registration of research studies

In accordance with the Declaration of Helsinki 2013, all research involving human participants has to be registered in a publicly accessible database. Please enter the name of the registry and the unique identifying number (UIN) of your study.

You can register any type of research at http://www.researchregistry.com to obtain your UIN if you have not already registered. This is mandatory for human studies only. Trials and certain observational research can also be registered elsewhere such as: ClinicalTrials.gov or ISRCTN or numerous other registries.

Not appliable
